# It does matter where you come from: mothers’ experiences of childbirth in midwife obstetric units, Tshwane, South Africa

**DOI:** 10.1186/s12978-017-0411-5

**Published:** 2017-11-16

**Authors:** Sarie J. Oosthuizen, Anne-Marie Bergh, Robert C. Pattinson, Jackie Grimbeek

**Affiliations:** 1 Tshwane District Health and Department of Family Medicine, University of Pretoria, Pretoria, South Africa; 2 South African Medical Research Council Unit for Maternal and Infant Health Care Strategies, University of Pretoria, Pretoria, South Africa; 3 Department of Obstetrics and Gynaecology, University of Pretoria, Pretoria, South Africa

**Keywords:** Respectful maternity care, Midwife obstetric units, Predictors of respectful care, Childbirth, South Africa

## Abstract

**Background:**

Health professionals are striving to improve respectful care for women, but they fall short in the domains of effective communication, respectful and dignified care and emotional support during labour. This study aimed to determine women’s experiences of childbirth with a view to improving respectful clinical care practices in low-risk, midwife-led obstetric units in the Tshwane District Health District, South Africa.

**Methods:**

A survey covering all midwife-led units in the district was conducted among 653 new mothers. An anonymous questionnaire was administered to mothers returning for a three-days-to-six-weeks postnatal follow-up visit. Mothers were asked about their experiences regarding communication, labour, clinical care and respectful care during confinement. An ANCOVA was performed to identify the socio-demographic variables that significantly predicted disrespectful care. Six items representing the different areas of experience were used in the analysis.

**Results:**

Age, language, educational level and length of residence in the district were significantly associated with disrespectful care (*p ≤* 0.01). Overall, the following groups of mothers reported more negative care experiences during labour: women between the ages of 17 and 24 years; women with limited formal education; and women from another province or a neighbouring country. Items which attracted fewer positive responses from participants were the following: 46% of mothers had been welcomed by name on arrival; 47% had been asked to give consent to a physical examination; and 39% had been offered food or water during labour. With regard to items related to respectful care, 54% of mothers indicated that all staff members had spoken courteously to them, 48% said they had been treated with a lot of respect, and 55% were completely satisfied with their treatment.

**Conclusion:**

There is a need to improve respectful care through interventions that are integrated into routine care practices in labour wards. To stop the spiral of abusive obstetric care, the care provided should be culturally sensitive and should address equity for the most vulnerable and underserved groups. All levels of the health care system should employ respectful obstetric care practices, matched with support for midwives and improved clinical governance in maternity facilities.

**Electronic supplementary material:**

The online version of this article (dio: 10.1186/s12978-017-0411-5) contains supplementary material, which is available to authorized users.

## Plain English summary

Although health professionals are aware of disrespect and abuse of women in labour, they fail to provide respectful maternity care. This study aimed to assess women’s experiences of and satisfaction with childbirth in low-risk, midwife-led obstetric units in the Tshwane District, South Africa.

A survey covering all 10 midwifery units in the district was conducted among 653 new mothers. An anonymous questionnaire was administered to mothers returning for a three-days-to-six-weeks postnatal follow-up visit. Mothers were asked about their experiences regarding communication, labour, clinical care and respectful care during confinement.

Only 48% of mothers felt that they had been treated with a lot of respect, while 55% of the respondents were satisfied with their treatment during confinement. The socio-demographic variables of age, language, educational level and length of residence in the district were significantly associated with disrespectful care (*p* ≤ 0.01). The following vulnerable groups reported significantly greater mistreatment in these areas: teenagers and young adults, women with limited formal education, women who do not speak the dominant language of the area as their first language, and women residing in the district for under 20 years.

Quality improvement approaches should recognise the plight of vulnerable women and accommodate them in respectful routine care practices in district labour wards. The care should be culturally sensitive and interventions should address equity for these vulnerable groups. All levels of the health care system should activate respectful obstetric care practices, matched with support for midwives and improved clinical governance in maternity facilities.

## Background

Globally, health professionals are striving to improve respectful care for pregnant women and birthing mothers within the limitations of their countries’ health systems. The Quality of Care Framework for maternal and newborn health of the World Health Organization (WHO) identifies the following domains of care: effective communication, respectful and dignified care and emotional support to improve women’s experiences of care during childbirth [1, 2]. Although mothers’ perspectives on quality care and the clinical outcome they experience should not differ from the aspects valued by health professionals, the literature highlights how divergently aspects of respectful professional care can be interpreted [1, 3]. Improved care for birthing mothers implies working with women to obtain their perspective on what constitutes a positive experience during labour and quality maternity services [4]. Frontline professional nurses and midwives play a key role in providing acceptable primary health care services to the public, as client satisfaction is mostly determined by their attitude and behaviour [5].

In South Africa and other low- and middle-income countries (LMICs) many patients with low-risk pregnancies still prefer a hospital delivery where there is a doctor present, bypassing the primary health care midwife-led obstetric units (MOUs) or community health centres and overloading the delivery units in hospitals [6, 7]. Many studies have explored care processes and described the way mothers would feel about care that responded to basic human needs and to cultural diversity [8–11]. Addressing most of the mothers’ reasonable preferences would improve satisfaction, as well as the quality of maternity care [12, 13] and would help to promote women’s willingness to deliver at lower levels of care [14].

Various studies explored the acceptability of obstetric care and barriers to access and use of maternal health services in South Africa [15]. Abusive obstetric practices in South African maternity facilities have been described as a “disgrace” [10] and a human rights violation impacting on autonomy, privacy, physical and psychological integrity, dignity and equality [16]. Calls have been made to address this important dimension of violence against women [10, 12, 17].

In the literature disrespectful obstetric care is described by a range of overlapping terms. Bohren et al. propose that a standardised typology be adopted to inform research and measurement tools [5]. For the purpose of this paper we adopted the term “respectful care”, which includes mothers’ report on specific labour practices and their experiences of and satisfaction with the care received. Although it was not included in our study, health-systems factors also impact on the ability to provide respectful care.

The measurement of birth satisfaction is complex and multifaceted, with women constructing the experience on the basis of their background and beliefs. Their experiences include the outcome of their labour, communication practices and the sharing of decisions made during the process of labour, as demonstrated by some birth satisfaction scales and questionnaires [18, 19]. Measuring different aspects of respectful care during labour would ensure that the projected improvements in care are balanced against the individual patient’s culture and social context and the specific needs of the birthing mother [20, 21]. Unequal treatment during childbirth and abuse of patients, as well as inequalities in service delivery need to be improved after measurement [15, 17].

The aim of our study was to assess women’s experiences of respectful care during childbirth and the early postnatal period in the Tshwane District, South Africa. The study formed part of the baseline assessment in the first phase of a larger interventional study conducted in the Tshwane District in 2016 to improve respectful clinical care practices in MOUs. The overall study was approved by the Research Ethics Committee of the Faculty of Health Sciences, University of Pretoria (Protocol 541/2015) and the Tshwane District Research Committee. Written permission was also obtained from the facility managers of all participating MOUs.

### Research setting

The Tshwane District Health Service provides health care to a population of 3.3 million and is categorised as one of the least deprived districts in South Africa, ranking in the top socio-economic quintile. The district recorded around 50,000 deliveries per annum in public facilities in the years 2014 and 2015. During the same period the district recorded a delivery-in-facility rate of 96.7% [22]. Around 18% of the district deliveries took place in the 10 MOUs and another 13% of women in labour were transferred from these MOUs to hospitals for care during delivery [22].

MOUs are located in either community health centres or larger clinics and attend to low-risk deliveries as part of the free primary health care system in South Africa. MOUs are able to provide basic emergency obstetric care [23], except for the removal of retained products of conception and assisted deliveries. Seven of the Tshwane MOUs are situated in urban areas, and 3 units are based in semi-rural settings. The latter facilities are located much further from referral sites offering caesarean sections (up to 70 km), with ambulance turn-around times of 1 h and longer. At the time of the study the midwife teams in each MOU consisted of two to four midwives per shift, depending on the facility’s available human resources and the number of deliveries per month. Each shift had a midwife specialist or advanced midwife on duty, who holds an additional qualification in midwifery and is registered with the South African Nursing Council [24].

In 2014, the South African National Department of Health launched the MomConnect mHealth initiative using mobile phones to register pregnancies and interact with the registered women, with a opt-in platform that encourages women to rate the services at the facilities [25]. Tshwane District received 63 antenatal-care-related or drug-related complaints from mothers between 2014 and 2016, but no complaints regarding mothers’ intra-partum care or narratives of distress.

## Methods

A baseline survey was conducted in all 10 MOUs in the Tshwane District from February to April 2016, to explore women’s experiences of childbirth and early postnatal care. A survey method was considered an appropriate methodology for measuring maternal experiences to gauge respectful care, involvement in decision making and clinical care processes [26].

### Survey tool

Data were collected by means of an anonymous, self-administered questionnaire with 32 structured and open-ended questions of which seven were socio-demographic items. The survey tool elicited data on the main concepts of respectful care. Sixteen items reflected women’s’ self-report of the clinical care they received and their experience thereof. The nine items on client satisfaction included aspects of communication and satisfaction. Items all required a yes/no/unsure response or a response on a four-point Likert-type survey scale. Many of the questions included in our survey had been used in previous sets of validated maternal experiences surveys and covered domains related to the lack of consented care, communication and feedback processes, pain relief and respectful care aspects [15, 18, 19, 27]. Text boxes included after satisfaction questions allowed participants to supply feedback and descriptions of poor service or abusive behaviour and to report unfulfilled expectations. The questionnaire was also made available in Setswana, the predominant local language, after translation from English to Setswana, followed by back-translation into English and the resolving of interpretation issues. The questionnaire was pilot-tested with 30 mothers to confirm the appropriateness of questions and ease of comprehension. (The questionnaire is attached as Additional file 1).

### Sample

The design of the sample for this survey was based on historical population data on annual deliveries at each MOU (range: 390 to 1502 in 2015). A planned sample of 800 respondents was envisaged, but 653 questionnaires were received back. Factors that impeded data collection included an unexpected drop in the number of deliveries in the district during the first 3 months of 2016 and several external service-delivery strikes that hampered access to the semi-rural areas.

### Data collection

Mothers completed questionnaires during the same period at various sites. University students fluent in the local vernaculars were trained as research assistants. They signed a confidentiality clause and assisted mothers with the completion of the questionnaire, only on request, in a private space. A sequential sample was drawn consisting of mothers returning for follow-up visits to primary health care consultation rooms in the three-days-to-six-weeks postnatal period. This gave mothers enough time to reflect on their care. Data were collected at a venue separate from the labour ward to minimise potential interference from MOU staff [28]. All mothers who had read the information leaflet and were willing to participate in the study were screened for eligibility. No mothers eligible for inclusion refused to participate. To qualify for inclusion, a mother had to be older than 17 years, have delivered in one of the 10 Tshwane MOUs and have returned for her postnatal visit during the prescribed period. Mothers younger than 17 years or those who had delivered in a hospital were excluded. Mothers completed the questionnaire voluntarily and anonymously.

### Data management and analysis

The completed questionnaires were collected, reviewed and coded. Data were captured in password-protected Excel files. The data were then crosschecked, cleaned and analysed with the aid of SAS version 9.4 [29].

Descriptive statistical measures such as means, frequencies and proportions were calculated and age, level of education, language, province or country of birth and length of residence in the Tshwane District were categorised to facilitate data interpretation. In order to establish the significance or importance of factors and attributes or items in the study that contributed to the acceptability or unacceptability of the treatment of mothers in the MOUs during childbirth, an analysis of variance approach was followed, with the inclusion of a covariate (ANCOVA). Due to the complexity of the analyses (multiple effects/factors), the classical approach of applying non-parametric procedures to ordered data, was not followed, but a transformation of the data as described below. For purposes of comparison, weighted means according to the number of 2015 deliveries based on scores on a Likert-type scale were calculated for categories of socio-demographic variables, namely age of mother, level of education, first language, province or country of birth, and length of residence in Tshwane. Missing values of categorical variables were replaced by hotdeck imputation [30], using simple random sampling with replacement of the units to produce complete data for a multivariate ANCOVA. The number of children a woman had given birth to was used as the covariate. The following six items relating to mothers’ experiences during childbirth were selected as dependents and a series of ANCOVAs were performed:

A : Did a member of staff attend to you within 15 min of arriving at the ward or unit?
B : Did the sister ask if it was okay to examine you?
C : Did any staff member say anything that upset you?
D : How did the staff speak to you during labour?
E : How respectfully do you think the sisters treated you during your stay in the labour ward?
F : How satisfied were you with the treatment you received in the labour ward?

The items were generally transformed from nominal [Yes, Unsure, No] measures to a Likert-type [0, 1, 2] scale. The ties present in the Likert scores were resolved by adding a small random univariate term from the [-0.000005; 0.0000005] interval and the resultant values were then normalised using the BLOM transformation [31]. Means for each item were calculated with scores of dependents as follows: Yes = 0, Unsure = 1, No = 2. The higher the means, the less positive the experience of the mother on an aspect of care; the lower the means, the more positive the experience. Means for a response were calculated for each of the categories of the selected socio-demographic variables, as well as the standard error based on this specific response for all categories of the particular demographic variable under consideration. The category corresponding to the maximum mean and all categories within one standard error of this maximum mean were considered as those contributing to the worst treatment of mothers in MOUs (see Additional file 2).

Open-ended responses related to the six items mentioned above were collated and some striking statements were selected to illustrate a particular issue.

## Results

### The sample obtained

The questionnaire was completed by 653 mothers. The number of respondents per MOU ranged from 29 to 102, sampled in accordance with the number of deliveries performed in 2015. Table 1 contains a summary of the socio-demographic characteristics of respondents of the 10 MOUs. Their mean age was 27.0 years, with half the respondents in the age category 25 to 34 years. The mean number of children per mother was 2.1. More than half of the mothers (51.6%) originally came from other provinces or neighbouring countries. Many of the respondents (64.3%) had been living in Tshwane for 5 years or more, while 13.0% had been in the district for less than 1 year. Some South African first languages were classified into a single cluster according to mutually understood language families, namely Sotho and Nguni. Two unrelated local African languages, Xitsonga and Tshivenda, were clustered together because they are spoken in neighbouring areas in the north of the country (Limpopo Province). The remaining two official South African languages, Afrikaans and English, were conveniently clustered together. Almost all mothers had some school education; over 50% had completed Grade 12 and some also had post-school education.

**Table 1:** Socio-demographic characteristics of respondents *(N* = 653*)*

Indicator	Categories	Frequency	Percentage^a^
Age range	Teenagers: 17–19 years	50	7.7
	Young mothers:20–24 years	201	30.8
	Adult mothers: 25–34 years	334	51.1
	Older mothers: 35 and above	66	10.1
	Unknown	2	0.3
Province/Country of birth	Gauteng Province	315	48.2
	Limpopo and Mpumalanga provinces	148	22.7
	Other provinces (Eastern Cape, Free State, KwaZulu-Natal, North West, Western Cape)	49	7.5
	Neighbouring countries (Zimbabwe, Mozambique, Malawi and other)	140	21.4
	Unknown	1	0.2
Living in Tshwane	Temporary: year <1	85	13.0
	Short term: 1 ≤ year <5	118	18.1
	Medium term: 5 ≤ year <20	194	29.7
	Long term: 20 ≤ year ≤ 45	226	34.6
	Unknown	30	4.6
Languages	Sotho (predominantly Setswana)	267	40.9
	Nguni (isiZulu, isiNdebele, isiXhosa, Seswati)	111	17.0
	Other local languages (Xitsonga, Tshivenda)	107	16.4
	Non-local African languages	132	20.2
	English, Afrikaans	28	4.3
	Unknown	8	1.2
School education	No school and primary education (Gr 0–7)	52	8.0
	Grades 8–11	240	36.8
	Grade 12 and post-school	346	53.0
	Unknown	15	2.3

^a^Raw percentages (no weighting)

### Experiences during childbirth with weighted results

Respondents spent a mean time of 7.0 h in the MOU before their babies were born. Table 2 gives an overview of mothers’ experiences of childbirth in different areas: communication, labour experiences, clinical care and perceptions of respectful care. Midwives greeted less than half of women in labour by name when receiving their maternity file on arrival at a facility. Consent to perform a physical examination was obtained from less than half of the women. Only 18.6% of respondents who requested this were allowed or offered a birthing partner during labour; 58.7% indicated that they preferred not to have a family member or partner with them because of current cultural custom. Around half of the mothers noted that all staff spoke to them courteously (“nicely”) (54.3%) and treated them with a lot of respect (47.9%), and 54.9% of mothers were completely satisfied with their birthing treatment.

**Table 2:** Mothers’ experiences during childbirth *(N* = 653*)*

Areas of mothers’ responses	Frequency	Weighted percentage
Communication with mothers:		
Attended by nurse within 15 min of arrival	504	74.5
Greeted by name on arrival	308	45.7
Asked how the woman was doing	498	73.7
Staff members addressed woman in a language she understood	583	89.3
*All staff members*	*473*	*69.9*
*Some staff members*	*110*	*19.4*
Labour experiences:		
Received information on progress	462	69.8
Consent given for examination	299	47.1
Vaginal examination done gently	514	77.6
Delivery attended by a staff member	595	91.2
Midwife who did the delivery introduced herself	238	36.3
Food, water or drinks offered while in labour	241	39.4
Labour experiences sub-groups:		
Wanted support from family member or partner during labour	253	41.3
*Staff offered/allowed a family member or partner support (n =* 253*)*	*43*	*18.6*
Clinical care:		
Hand washing observed	331	50.1
Skin-to-skin contact with the baby directly after birth	511	79.0
Information given on baby’s care	540	84.4
Clinical care sub-groups:		
Complications or problems during birth	49	8.2
*Health professional blamed mother for complications (n =* 49*)*	*7*	*14.4*
Help needed with breastfeeding	629	96.7
*Not helped with feeding when needed (n =* 629*)*	*115*	*17.9*
Respectful care:		
All or some staff spoke nicely to woman during labour	555	85.2
*All staff spoke nicely*	364	54.3
*All or some staff were rude*	96	14.7
Staff said something upsetting	105	16.1
Mother’s perception of being treated respectfully (*n* = 647)		
*Treated with a lot of respect*	*307*	*47.9*
*Treated with some respect*	*263*	*40.6*
*Treated with little or complete disrespect*	*77*	*11.4*
Mother’s satisfaction with treatment (*n* = 653)		
*Completely satisfied*	*361*	*54.9*
*Somewhat satisfied*	*224*	*34.4*
*Somewhat or completely dissatisfied*	*68*	*10.7*

Pain relief medication was not available in four of the five MOUs during the study. One MOU with 102 respondents had pain medication on hand. It was offered and administered to 58 women (56.9%) in the sub-sample.

### Predictors of disrespectful care

**Fig. 1:**
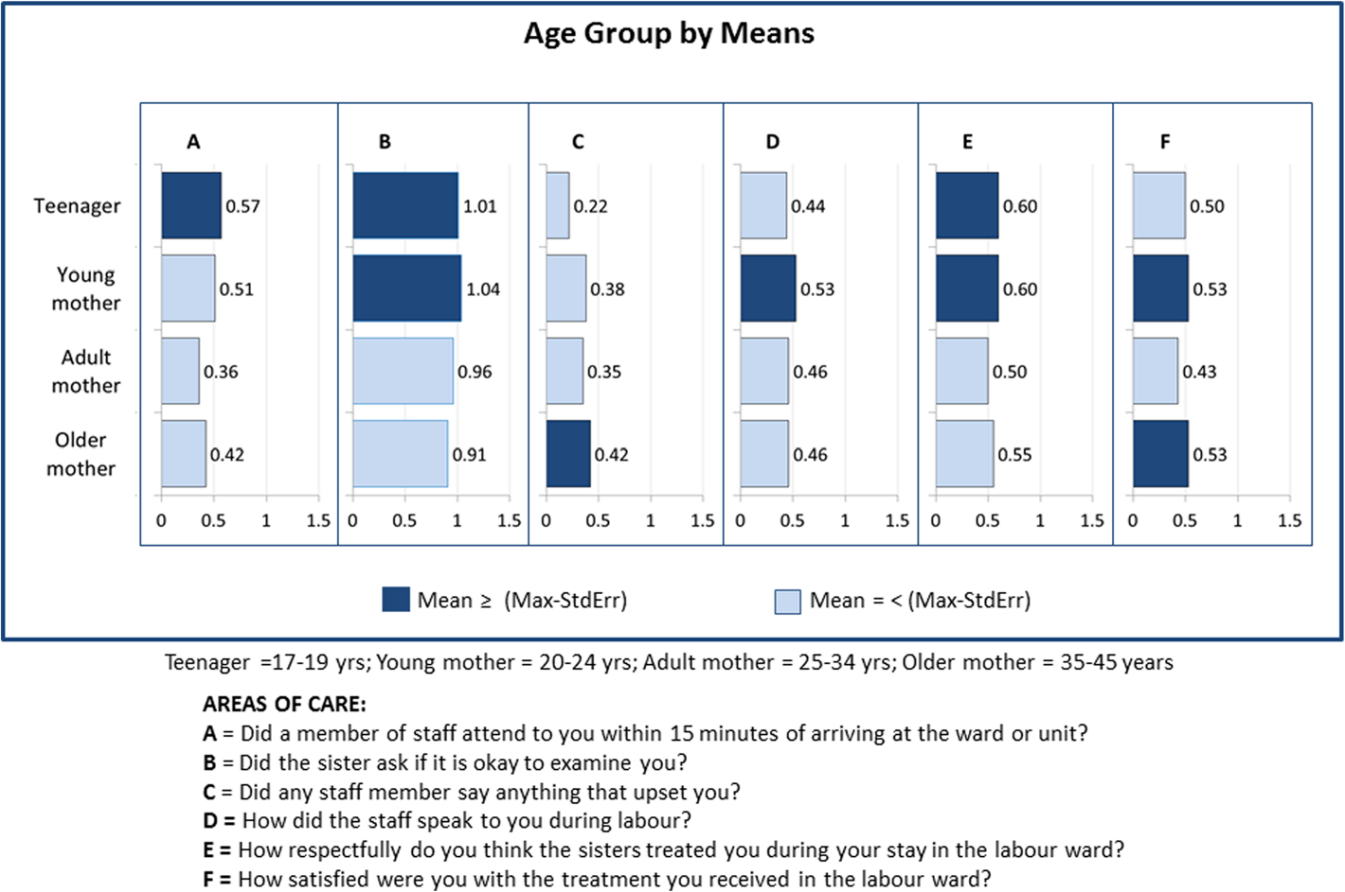
Example of mean response for a category of a significant socio-demographic variable (age)

Some socio-demographic variables were strongly linked to shortcomings during women’s childbirth and treatment. Table 3 visualises data – according to different levels of significance (*p =* <0.5; <0.1; <0.01) – on the respondent groups that reported the worst treatment for the six items selected for analysis. (For the results of the total analysis, see Additional files 2 and 3.) Fig. 1 provides an example of the calculation of the means of the class variable of age as a significant socio-demographic variable linked to the care women reported having received. The darker bars represent the categories contributing to the worst treatment of mothers, i.e. those categories within one standard error of the maximum mean. (Additional file 2 provides the same information in graphic form for all the variables.)

**Table 3:**
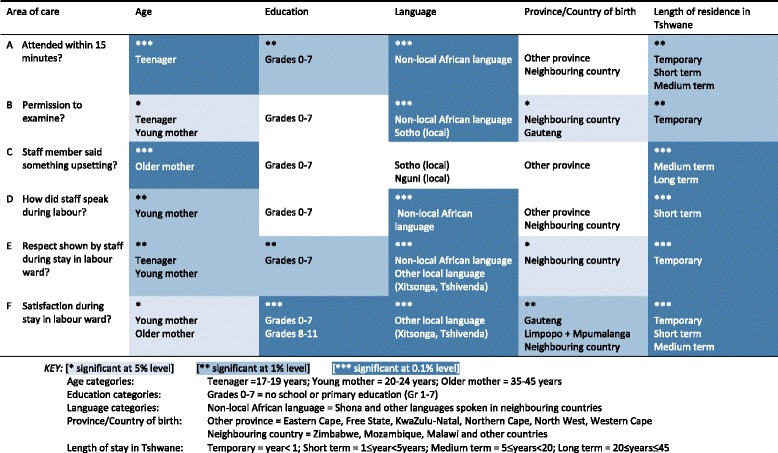
Respondent groups reporting poorer treatment during labour and more dissatisfaction with care

The following points summarise the main results for each of the six items (areas of care) included in the analysis:

A. Regarding being attended to within 15 min after arrival, teenage mothers and those from other countries experienced more delays, followed by those with a lower level of formal education and those who had been resident in Tshwane for a shorter period.
B. More mothers speaking a foreign language or one of the local Sotho languages, as well as mothers who had lived in Tshwane for less than 1 year, reported that they had not been asked for consent to a physical examination.
C. Older mothers and those who had been living in Tshwane for more than 5 years reported being more upset about something staff members had told them.
D. Mothers speaking a foreign language and those who had been living in Tshwane for between one and 5 years reported being spoken to more rudely, followed by young mothers in the age category 20–24 years.
E. Mothers with less than 1 year’s residence in Tshwane and those speaking a foreign language or Xitsonga or Tshivenda experienced less respectful care, followed by teenage and young mothers and those with a lower level of formal education.
F. The groups of respondents who were the most dissatisfied with their stay in the labour ward were those who had not completed their schooling (Grades 0–11), Xitsonga- and Tshivenda-speakers and those who had been living in Tshwane for less than 20 years. Foreign mothers and those from Gauteng, Limpopo and Mpumalanga also reported less satisfaction with their birthing experience.

Overall, the following groups of mothers reported to have had more negative experiences of treatment during labour: teenage and young mothers; mothers with no schooling or only primary education; mothers from other countries who speak a foreign language; and mothers with less than 20 years’ residence in Tshwane. Level of education and province or country of birth were the variables with the least influence on women’s experiences during childbirth. The results show that class variables of age, language and period of residence in Tshwane were significant predictors of the level of disrespect shown in communication and care and client dissatisfaction with treatment (*p =* <0.01), as seen in Table 3.

### Open-ended responses

Completion of open-ended responses varied from 41 to 403 for individual items. In total, only 55 (9%) of the total number of comments (*n =* 595) were positive. Several examples of poor communication, abusive and hurtful examinations, withholding of care and disrespectful care were given in these responses. Some of the marginalised mothers called for a change in midwives’ attitudes and compassion, requesting equal treatment:

*“We are also human!”.*

*“I am not happy about the way I had to deliver my baby!”.*

*“They must stop violence, treat us with respect, even if we are a teenage mother!”.*

*“They must stop telling mothers they are too young or too old to give birth”.*

*“They left me unattended and I gave birth on my own”.*

They described their experiences of physical abuse during examinations and delivery as follows:

“*They must be gentle when examining us, they don’t care whether they hurt us or not!*”

“*Sisters should make mothers aware that they are about to be examined – not to be penetrated with fingers while unaware!*”

*“They are harsh and refused to give me something for pain.”*

*“Some were annoyed with the mothers and ill-treating them.”*

*“They must stop the violence and not treat us like animals!”*

Some of the women took the opportunity to describe their observations of the health system and to complain about the physical structure of the MOUs and concomitant lack of privacy, lack of ablutions, dirty linen and lack of pest control in units, insufficient number of midwives in attendance per shift and occasional rudeness from support staff. They reported the mode of communication as *“forever shouting”*.

## Discussion

Our study explored respectful care in midwife-led obstetric units with reference to the following areas: the socio-demographic characteristics of clients arriving at an MOU; the welcome they received and their communication experiences; the processes of clinical care during childbirth; and measures of satisfaction and humane treatment. The use of a survey with structured and open-ended questions shed light on the domains of adequate clinical care and failure to meet the mothers’ needs and expectations. To our knowledge, this study is the first to measure women’s satisfaction with maternity care at primary health care level in the Tshwane District, South Africa. Our results provide further information on women’s experience of care during childbirth according the key domains of the WHO quality of care framework, namely communication, respect and dignity, and emotional support [1]. This could be considered a contribution to the development of innovative care tools to measure satisfaction as proposed in the “Passport to Safer Birth” [32, 33].

The findings in all Tshwane MOUs matched the disregarded shortcomings of disrespectful, abusive care and poor communication practices recorded in other LMIC countries, such as Tanzania, Ghana and Nigeria [34–36]. While violent abuse [36] was not reported as often in our study, many women complained about verbal abuse, the midwives’ attitudes and behaviour, abandonment and the fact that they did not receive care when needed. As in Nigeria [27], 54% of the clients of Tshwane MOUs reported non-consented care. Only 55% of Tshwane mothers were completely satisfied with their birthing experience, highlighting insensitivity to mothers’ birthing needs [5] and non-adherence to the WHO quality of care framework [1]. The National Department of Health launched the ideal clinic document and checklists in April 2017 [37], stating that patient experiences of care should be in line with the national core standards of health establishments in South Africa and should reach an overall score of 80% to be in the green zone [38]. While satisfaction with services is not easily measured or defined, women regard the birthing of a healthy baby as the end goal, accepting any spectrum of disrespect and abuse, as defined by Bowser and Hill [39]. A greater focus on ideal communication and empowering women to complain would remove “normalised disrespectful care” [40].

In our study, women’s age, language, educational level and period of residence in the Tshwane District were significantly linked to the midwife’s attitude, communication and caring behaviour, as was also highlighted in a review of the literature [41]. Any mother coming from a different culture, marginalised group or low socio-economic background can expect more abuse and disrespect, as documented in this study and other studies on maternal satisfaction [34, 41, 42]. The socio-demographic variables associated with the way midwives involve mothers during their welcome, the promptness of their clinical care and how respectfully they treat those mothers during delivery should inform strategies to strengthen the health system. Maternity care professionals and programme managers should highlight diversity and advocate equity for all vulnerable groups, with on-going monitoring and evaluation of respectful care in units [43–45], addressing the lack of accountability and inaction against abuse [11].

The proper welcoming of women is the first step in better communication, trust building and empathic care during childbirth [46], thereby addressing the human rights principle of dignity [47]. All MOUs performed poorly in welcoming their patients and greeting them by name on arrival. Discrimination, cultural insensitivity and disregard for non-local mothers’ wishes in health care settings have been widely reported in LMICs [27, 48, 49]. While birthing partners provide extensive benefits to the birthing mother and family unit [50], only 39% of women in this study would have preferred to have a partner present during the birth of their baby. This observation may reflect cultural barriers or lack of empowerment of women in their communities.

Although the proportion of women under the age of 18 delivering in Tshwane decreased to a rate of less than 5% in 2015 for delivery-in-facility by mothers under the age of 18 years [22], the effect of their age and education on the birthing care they receive remains a matter of concern to health managers. Younger mothers with a lower level of formal education and those who hail from a different cultural background and speak a different language become an easy target in a resource-constrained health care system, as documented here and in other LMICs [17, 49]. Clinical care processes and pain relief were dependent on the skills and knowledge of the attending midwives, with many midwives lacking confidence in their ability to resuscitate neonates. Pain relief was available in only one MOU, which means that fewer than one in 10 women had access to pain relief. Health management systems and policy makers who are focusing on high-quality clinical care should ensure that humane clinical care and pain relief are once again embedded in routine birthing care, thereby improving respectful clinical care as envisaged in the BOLD study protocol [51].

This study, like many others, highlighted the lack of respectful care during childbirth, especially in LMICs, influenced by the attitudes and behaviours of maternal health professionals [20], interlinked with contextual factors in the health system [12, 52, 53] and socio-demographic characteristics of the mothers [5, 54].

### Study strengths and limitations

A strength of this study is the fact that all 10 MOUs in one district were surveyed regarding experiences of care during childbirth. The limited period of 9 weeks for data collection provided the opportunity to obtain a comprehensive snapshot of care experiences during labour. The open-ended questions collected responses to clarify aspects of dissatisfaction and abuse in MOUs.

Limitations of the study relate to logistics that constrained data collection, including distances between MOUs, as well as external service-delivery protests, which limited access to some MOUs on certain days. A sequential sample is a form of convenience sampling, so only limited claims can be made with regard to generalisation and representativeness. Although our survey was conducted outside labour wards, fear and mistrust of the providers could have influenced respondents’ recall of negative events or triggered the coping mechanisms they employed to protect themselves from recalling the birthing.

## Conclusion

“It does matter where you come from” has shown that equity for the most vulnerable groups in district health services should be attained by emphasising the risk of delays in or withholding of clinical care, denigrating communication, abusive and hurtful examinations, and disrespectful care of the younger and older mother, mothers from other countries and those speaking a foreign language, mothers from minority groups within the country and mothers with lower levels of formal education. This goal can only be achieved if obstetric care of high quality is offered in MOUs. Interventions should address changes in the context of respectful relationships and dignity, effective communication and emotional support to improve the childbirth experience in labour wards. An intervention package is needed that would enable respectful obstetric care on the micro-, meso- and macro-levels of the health care system, matched with support to midwives and local accountability in birthing facilities.

## Additional files

Additional file 1: Anonymous questionnaire for mothers of newborn babies. (PDF 78 kb)

Additional file 2: ANCOVA significant differences of class variables and graphs of maximum of means within standard error of maximum mean. (PDF 622 kb)

Additional file 3: Results of ANCOVA with observations weighted according to the number of deliveries. (PDF 378 kb)

## Abbreviations

LIMC: Low- and middle-income country
MOU: Midwife obstetric unit
SDG: Sustainable development goal
