# Supplementary material for: It does matter where you come from: mothers’ experiences of childbirth in midwife obstetric units, Tshwane, South Africa
Source: Reprod Health. 2017 Nov 16;14:151. doi: 10.1186/s12978-017-0411-5 (PMC5689145; doi:10.1186/s12978-017-0411-5)
Supplement: Supplementary file 1 — Anonymous questionnaire for mothers of newborn babies. (PDF 78 kb) [file 12978_2017_411_MOESM1_ESM.pdf]

Additional file 1 Number: ________ Date completed: ____________________

ANONYMOUS QUESTIONNAIRE FOR MOTHERS OF NEWBORN BABIES

[Cover letter can be adapted according to researcher's needs and the research context]

You are invited to volunteer to participate in our study. We want to find out more about the treatment you received at the clinic when you gave birth to your baby/babies. This information is important for us to be able to improve the services that patients receive.

This letter gives information to help you to decide if you want to take part. We would like you to complete a questionnaire. This may take about 10 to 15 minutes. A health worker will take the questionnaire from you after you have filled it out. Please do not write your name on the questionnaire. We will keep it in a safe place to ensure confidentiality and no one at the clinic will see your answers. We will be available to help you with the questionnaire or to fill it in on your behalf.

[Information on ethics approval]

It is voluntary to take part in this study. You don't need to answer all the questions if you don't want to and you can refuse to take part or stop at any time without giving any reason. You do not write your name on the questionnaire and you give us the information anonymously. Once you have given the questionnaire back to us, we will not know it is your information and cannot give the questionnaire back to you. This means that you gave us informed consent that we may use the information. Because you give the information anonymously, you cannot be identified in any publication.

If there is anything in the questionnaire that upsets you or make you sad, we can get someone to talk to about it if you wish.

We sincerely appreciate your help.

We would like to get a few personal details about yourself.

1. At which clinic was your baby born?

Dark City	Eersterust	Kgabo	Laudium	Stanza CHC
Boekenhout	Temba	Phedisong 4	Refentse	Soshanguve

2. How old are you?

I am __________ years old	OR	My date of birth is _dd / mm / yyyy

3. How many children have you given birth to?

Number of children:_______	Number of stillborn babies:_______

4. What is the highest level of school education that you completed?

No school	Gr 0-7	Gr 8	Gr 9	Gr 10	Gr 11	Gr 12	Other (complete) ___________________

5. Where were you born?

Gauteng	Mpumalanga	Limpopo
Free State	North West	Eastern Cape
KwaZulu-Natal	Northern Cape	Western Cape
In another country (complete) ____		

6. If you are living in Tshwane, for how long?

_________months OR _________ years	Not living in Tshwane

7. What is your first language?

Setswana	Sepedi	Sesotho	Afrikaans
isiZulu	isiNdebele	SiSwati	isiXhosa
Tshivenda	Xitsonga	English	
Other (complete) ____			

We also want to know more about your experience of the labour ward and the birth of the baby.

8. Did a member of staff attend to you within 15 minutes of arriving at the ward or unit?

Yes	No	Unsure

9. Did a member of staff greet you by name when you arrived at the labour ward?

Yes	No	Unsure

10. Did a member of staff ask you how you were doing?

Yes	No	Unsure

11. Did any of the staff members looking after you speak to you in a language that you understand?

All staff members	Some staff members	No staff members

12. How many hours were you in the clinic before the baby was born?

_________ hours	Don’t know

13. Were you offered or given tablets or an injection for pain while you were in labour?

Yes	No	Unsure

14. Did the staff tell you that you may have a family member or partner with you during labour and birth of the baby?

Yes	No	Unsure

15. Did the sister ask if it is okay to examine you?

Yes	No	Unsure

16. Did the sister try to be gentle during physical examinations, not causing too much pain?

Yes	No	Unsure

17. Did any member of staff offer you any food or drinks while you were at the clinic?

Yes	No	Unsure

18. Did any of the sisters tell you how you and the baby are doing during labour and delivery?

Yes	No	Unsure

19. Did any staff member say anything that upset you?

Yes	No	Unsure
If Yes, can you give an example? ______________________________		

20. Did the sister who delivered your baby, introduce herself?

Yes	No	Unsure

21. Was there any staff member with you when the baby was born?

Yes	No	Unsure

22. Did the staff ignore you when you asked for help?

Yes	No	Unsure	Did not ask for help
If Yes, can you tell us what happened? ______________________________			

23. Did the staff call your family member or partner to be with you during the birth of the baby?

Yes	No	Unsure
.1 Did you want someone to be with you during the birth?		
Yes	No	Unsure

24. How did the staff speak to you during labour?

Mark ONE box that best describes how the staff spoke to you

All staff spoke nicely	Some staff were rude
Some staff spoke nicely	All staff were rude
Can you give an example? ______________________________	

25. Did you have any complications or problems during the birth of the baby?

Yes	No	Unsure
If Yes, please answer the following two questions:<br>.1 What was the complication or problem? ______________________________		
.2 Did the sister blame you for the complication or problem?		
Yes	No	Not applicable
Can you tell us what happened? ______________________________		

26. Did you see the sisters wash their hands before they touched you or the baby?

Yes	No	Unsure

27. After birth, did the sister put your baby naked on your tummy or chest, so that you had skin to skin contact with your baby?

Yes	No	Can't remember

28. Did the staff help you with breastfeeding?

Yes	No	Did not need help

29. Did the sisters give you information on the care of your baby before discharging you?

Yes	No	Can't remember

30. How respectfully do you think the sisters treated you during your stay in the labour ward?

Mark ONE box that best describes your impression

A lot of respect	Some respect	Little respect	No respect

31. How satisfied were you with the treatment you received in the labour ward?

Mark ONE box that best describes your experience

Completely satisfied	Somewhat satisfied	Somewhat dissatisfied	Completely dissatisfied
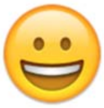	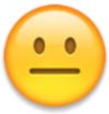	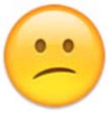	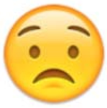

32. How do you think the sisters can improve the care of the mothers during labour?

Examples:

Thank you for completing this questionnaire
