# Supplementary material for: It does matter where you come from: mothers’ experiences of childbirth in midwife obstetric units, Tshwane, South Africa
Source: Reprod Health. 2017 Nov 16;14:151. doi: 10.1186/s12978-017-0411-5 (PMC5689145; doi:10.1186/s12978-017-0411-5)
Supplement: Supplementary file 2 — ANCOVA significant differences of class variables and graphs of maximum of means within standard error of maximum mean. (PDF 622 kb) [file 12978_2017_411_MOESM2_ESM.pdf]

Additional file 2: **ANCOVA significant differences of class variables and graphs of maximum of means within standard error of maximum mean**

Mean of Scores [0, 1, 2] Weighted by Deliveries of 2 Month period

[0 = Yes/Positive, 1 = Unsure/Undecided, 2=No/Negative] (n=653)

AREAS OF CARE:

A = Q08: Did a member of staff attend to you within 15 minutes of arriving at the ward or unit?

B = Q15: Did the sister ask if it is okay to examine you?

C = Q19: Did any staff member say anything that upset you?

D = Q24a: How did the staff speak to you during labour?

E = Q30: How respectfully do you think the sisters treated you during your stay in the labour ward?

F = Q31: How satisfied were you with the treatment you received in the labour ward?

Table

Class variable	Category	Sample size (n)	A	B	C	D	E	F
Age	Older mother	66	0.42	0.91	0.42	0.46	0.55	0.53
	Adult mother	336	0.36	0.96	0.35	0.46	0.50	0.43
	Young mother	201	0.51	1.04	0.38	0.53	0.60	0.53
	Teenager	50	0.57	1.01	0.22	0.44	0.60	0.50
Qualifications	Grade 12+	352	0.41	0.98	0.32	0.47	0.53	0.46
	Grade 8-11	249	0.42	0.96	0.39	0.46	0.56	0.49
	Grade 0-7	52	0.52	1.14	0.44	0.60	0.60	0.48
Province/Country of birth	Neighbouring country	140	0.55	1.03	0.32	0.57	0.63	0.47
	Other province	50	0.55	0.95	0.42	0.55	0.51	0.43
	Limpopo & Mpumalanga	148	0.38	0.91	0.31	0.48	0.52	0.49
	Gauteng	315	0.37	1.01	0.39	0.42	0.53	0.48
Length of stay in Tshwane	Temporary	90	0.48	1.05	0.32	0.49	0.64	0.48
	Short term	122	0.50	0.95	0.27	0.58	0.50	0.48
	Medium term	205	0.49	0.99	0.40	0.51	0.59	0.50
	Long term	236	0.28	0.98	0.38	0.37	0.48	0.44
First language	Non-local African	138	0.55	1.02	0.32	0.57	0.62	0.47
	Xitsonga & Tshivenda	107	0.33	0.96	0.34	0.50	0.61	0.55
	Nguni	111	0.44	0.90	0.40	0.40	0.46	0.44
	Sotho	269	0.38	1.02	0.38	0.46	0.52	0.47
	Western	26	0.40	0.88	0.17	0.42	0.49	0.42

Graph 1

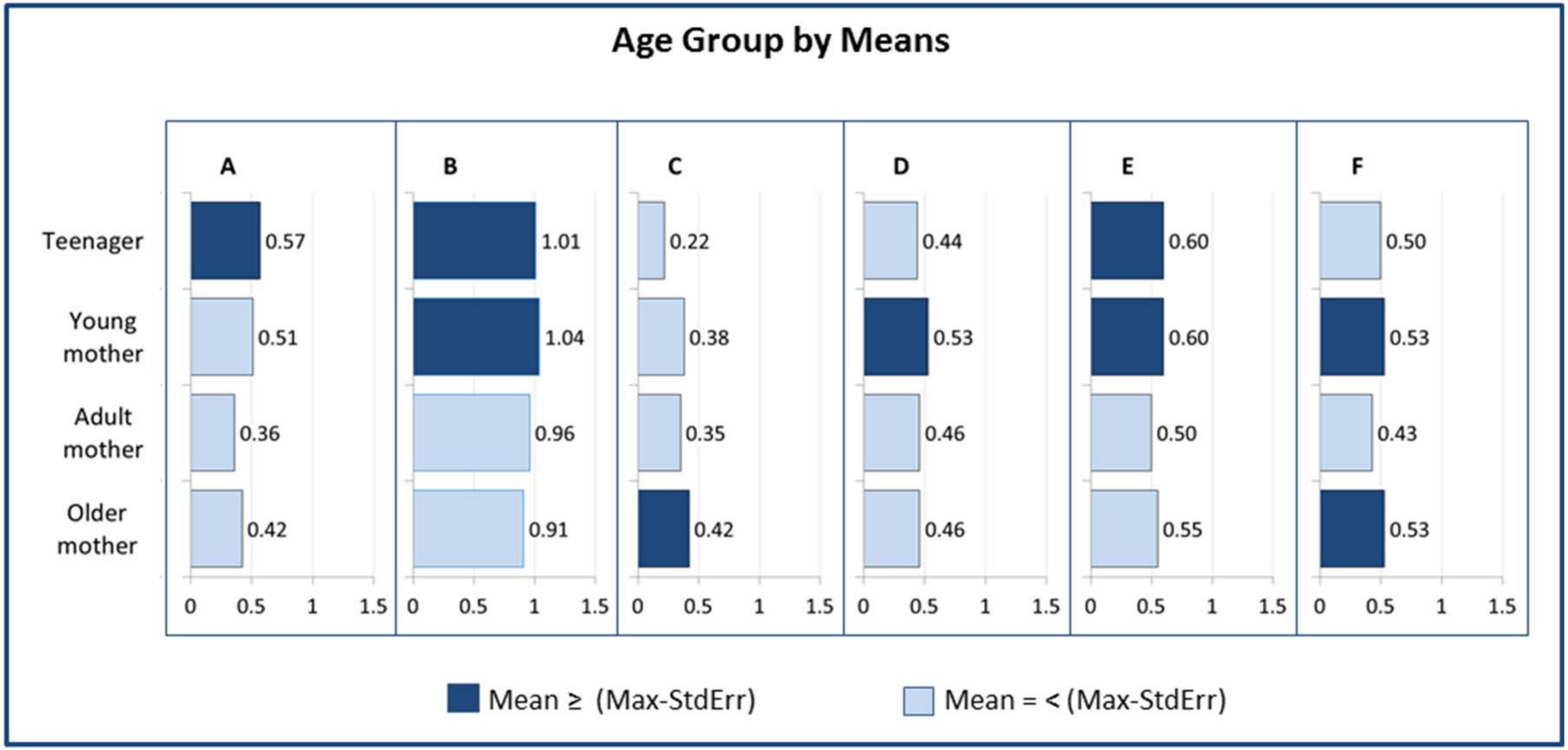

Teenager =17-19 yrs; Young mother = 20-24 yrs; Adult mother = 25-34 yrs; Older mother = 35-45 years

Graph 2

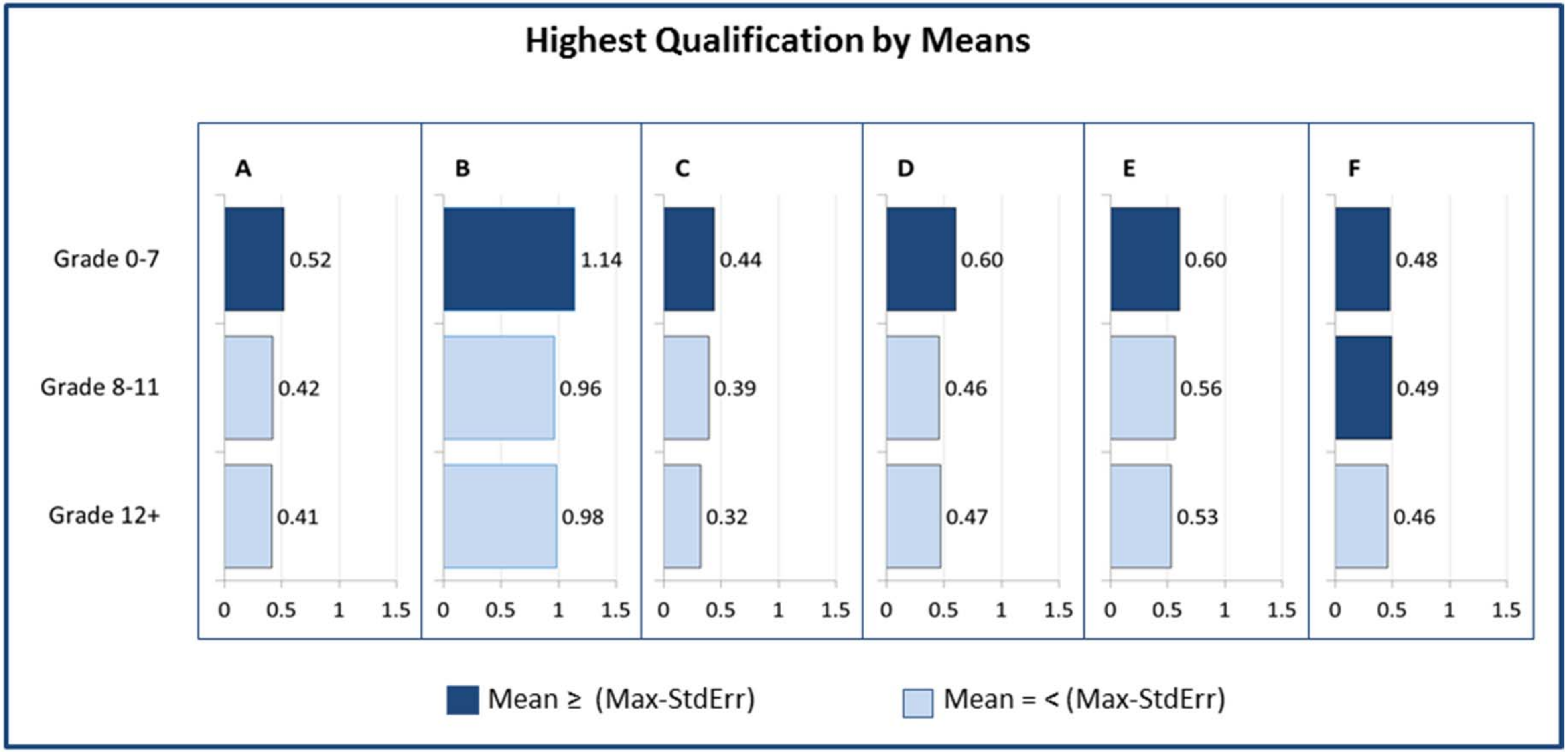

Grade 0-7 = no school or primary education; Grade 12+ = Grade 12 and post-school

Graph 3

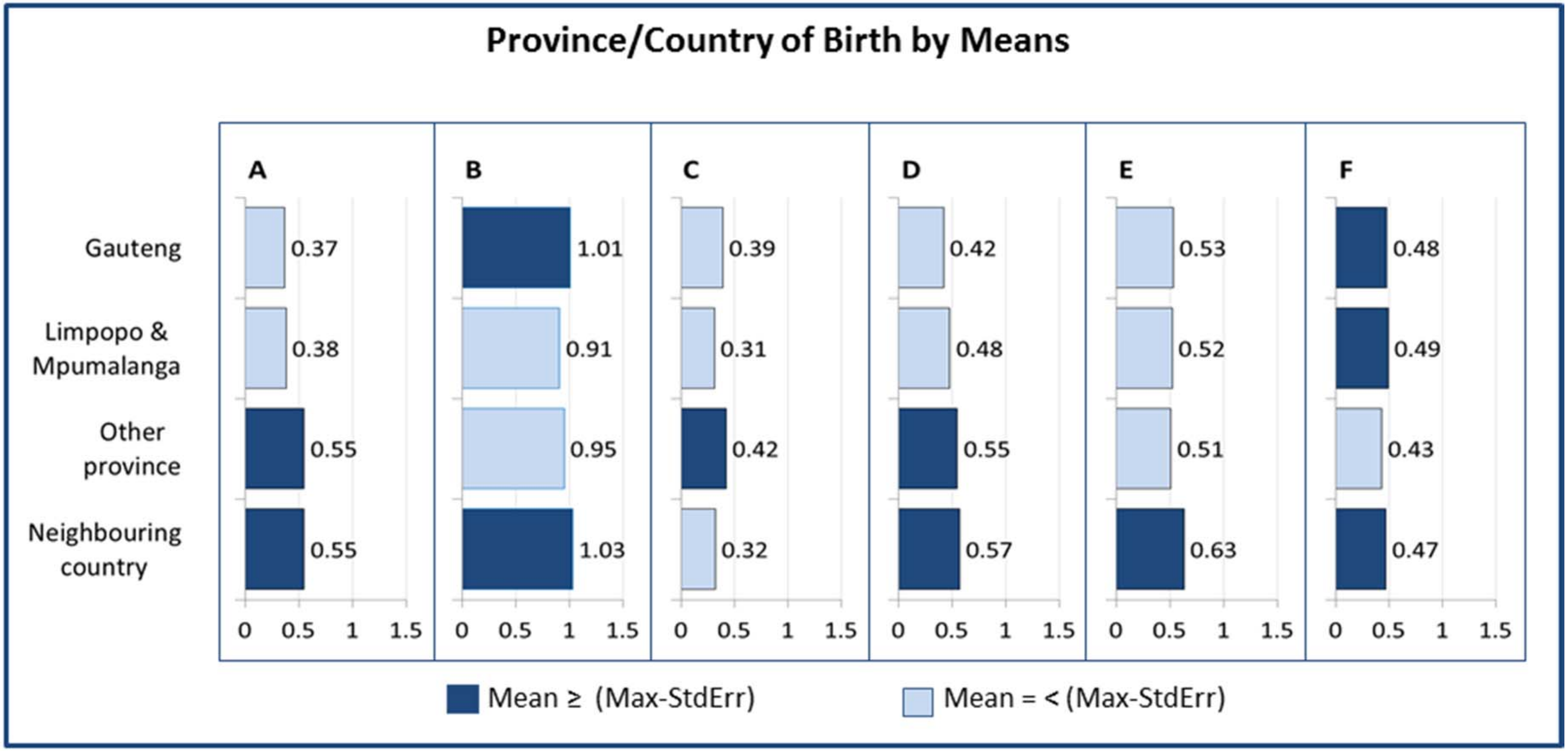

Other province = Eastern Cape, Free State, KwaZulu-Natal, Northern Cape, North West, Western Cape
Neighbouring country = Zimbabwe, Mozambique, Malawi and other countries

Graph 4

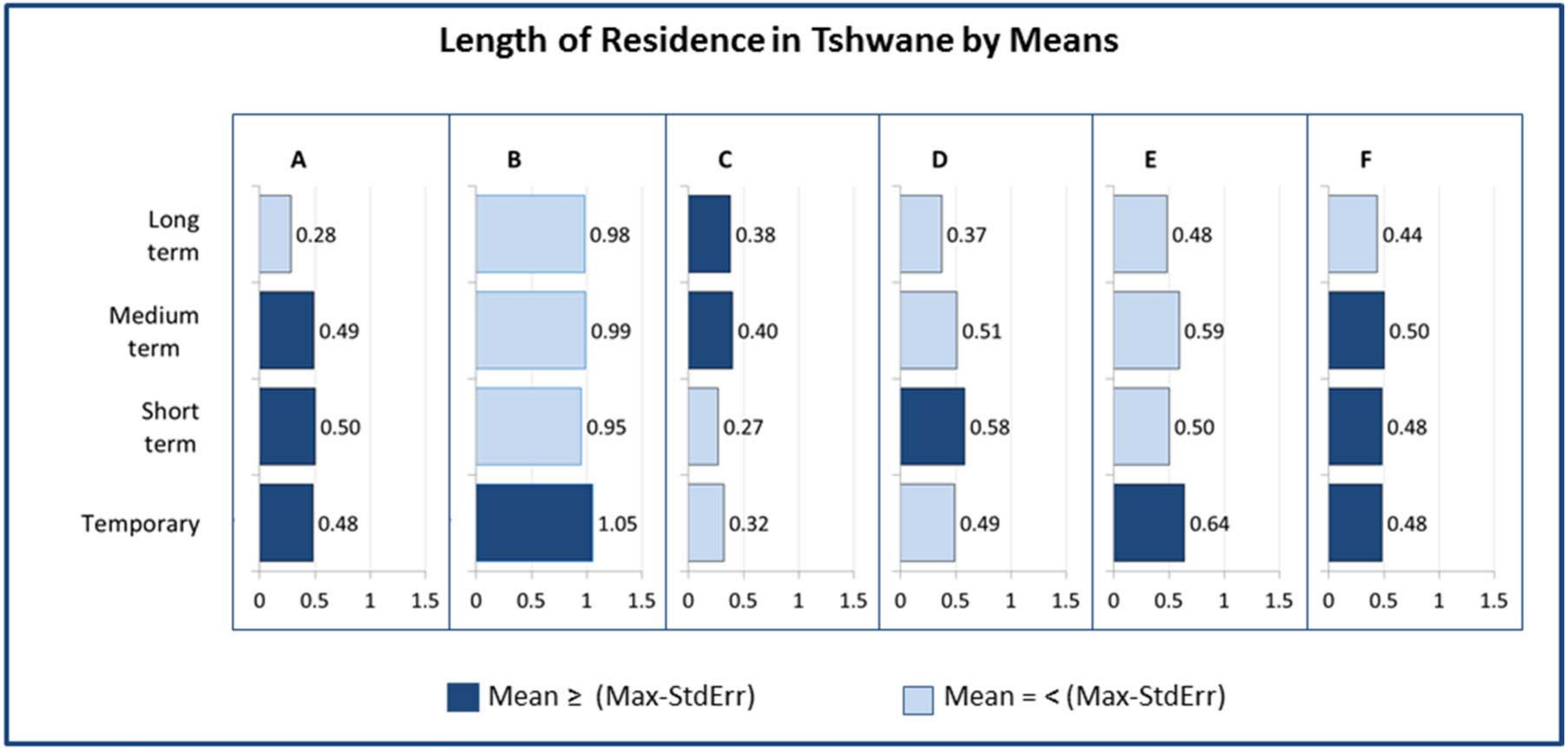

Temporary = year<1; Short term = 1≤year<5years; Medium term = 5≤years<20; Long term = 20≤years≤45

Graph 5

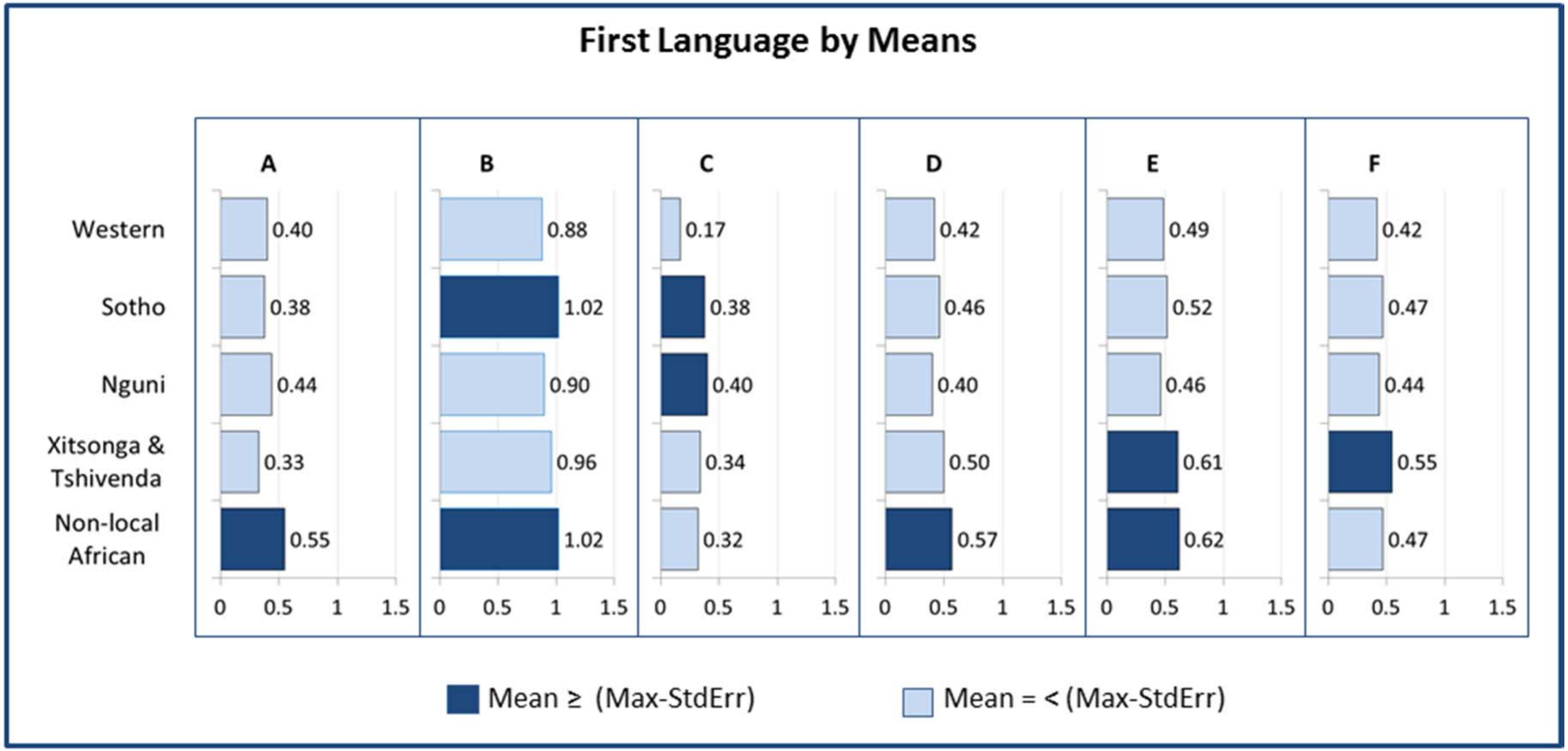

Western = English and Afrikaans; Non-local African = Shona and other languages spoken in neighbouring countries; Sotho = languages in the Sotho language family (Setswana, Sesotho, Sepedi); Nguni = languages in the Nguni language family (isiZulu, isiNdebele, isiXhosa, Seswati); Xitsonga & Tshivenda = other South African languages
