# Supplementary material for: It does matter where you come from: mothers’ experiences of childbirth in midwife obstetric units, Tshwane, South Africa
Source: Reprod Health. 2017 Nov 16;14:151. doi: 10.1186/s12978-017-0411-5 (PMC5689145; doi:10.1186/s12978-017-0411-5)
Supplement: Supplementary file 3 — Results of ANCOVA with observations weighted according to the number of deliveries. (PDF 378 kb) [file 12978_2017_411_MOESM3_ESM.pdf]

Additional file 3 Results of ANCOVA with observations weighted according to the number of deliveries (*N*=653)

Effect	Question[a]											
	Attended to within 15 min?		Permission to examine?		Staff member said something upsetting?		How did staff speak during labour?		Respect shown by staff during stay in labour ward?		Satisfaction during stay in labour ward?	
[Clinic] *(Stratification variable)*	<.0001[b]	***	<.0001	*** [c]	0.0047	**	<.0001	***	<.0001	***	<.0001	***
[Age Group]	0.0002	***	0.0451	*	0.0004	***	0.0060	**	0.0015	**	0.0234	*
[Age Group] x [Highest education level]	0.0001	***	0.0330	*	0.6427		0.1219		0.0088	**	0.4165	
[Age Group] x [Place of birth]	0.0404	*	0.4357		0.0002	***	0.4121		0.4169		0.0015	**
[Age Group] x [Length of residence in Tshwane]	<.0001	***	0.0004	***	0.0001	***	0.3053		0.0005	***	0.0530	
[Age Group] x [First language]	<.0001	***	<.0001	***	<.0001	***	0.0001	***	<.0001	***	0.0006	***
[Highest education level]	0.0022	**	0.2982		0.6340		0.3322		0.0021	**	0.0007	***
[Highest education level] x [Place of birth]	0.3792		0.0133	*	0.0123	*	0.4671		0.1474		0.0028	**
[Highest education level] x [Length of residence in Tshwane]	0.0640		0.0182	*	0.0178	*	0.2785		0.2116		0.0637	
[Highest education level] x [First language]	<.0001	***	0.1654		0.0010	***	<.0001	***	<.0001	***	0.0001	***
[Place of birth]	0.0742		0.0150	*	0.0805		0.6109		0.0222	*	0.0027	**
[Place of birth] x [Length of residence in Tshwane]	0.0890		0.2980		0.2751		<.0001	***	0.5991		0.8645	
[Place of birth] x [First language]	0.1419		<.0001	***	0.1914		<.0001	***	<.0001	***	<.0001	***
[Length of residence in Tshwane]	0.0099	**	0.0012	**	0.0006	***	0.0001	***	0.0004	***	0.0004	***
[Length of residence in Tshwane] x [First language]	<.0001	***	<.0001	***	<.0001	***	<.0001	***	0.0071	**	<.0001	***
[First language]	0.0004	***	0.0007	***	0.1538		<.0001	***	<.0001	***	<.0001	***
[Number of children already birthed] (Co-variate)	0.0143	*	0.3579		0.6494		0.5653		0.2808		0.9484	

a ≡ Questions recorded on a 3-point [0, 1, 2] Likert scale and normalised using the BLOM[36, 37] transformation
b ≡ p value = Exceedance probability of no significant difference
c ≡ [* significant at 5% level] [** significant at 1% level] [*** significant at 0.1% level]
